# An Analysis of Guislain's Work on Insanity

**Published:** 1854-07-01

**Authors:** 


### AN ANALYSIS OF GUISLAIN'S WORK ON INSANITY.

### Ninth Lecture.

(*Continued from No. XXVI., page* 273.)

*The complex forms of mania.*—Twenty-three forms of mania, without counting several compound forms not described. There is, you will say, perhaps, a symptomatological baggage sufficiently heavy for the memory. But you will perceive that to group thus the phenomena of the disease is to render its study more easy.

*Of general mania.*—In general mania, polymania, the sum of mental activity is doubled, multiplied tenfold; every act is an extravagance, an exaggeration, a passion. The patient who now serves as an illustration desires, wills, exacts; he wishes for a thousand different things at once; he complains of the limits set to his will. He wants to go out. He will not stay in a place where he says he is surrounded with enemies. He proposes to purchase a certain property; he wants to demolish this wall. In melancholy, as we have seen, the will is as if paralyzed; the moral in a state of prostration, at least unless the phrenalgia be associated with mania.

The morbid excitation invades the domain of the ideas. It is always some new plan, some new demand. A flood of projects is poured forth: the patient talks night and day. One might imagine a column of ideas escaping by a valve which had held it captive. The speech is clear. Error is at the bottom; but the form is traced with precision. The phrases are often incoherent. The words are sometimes only sounds without meaning, vociferations, or blasphemies.

In melancholy the patient accuses himself. In this maniac, on the contrary, the patient, far from accusing himself, is a victim. He believes himself surrounded with enemies, plots, and conspiracies. You may have remarked that the personal pronoun has been displaced in his case. It is not *I* am unfortunate, as with the melancholic, but *they* have a spite against me. The transition from melancholy to mania is announced by this change in the application of the personal pronoun.

Now observe the violence, the fits of anger, of fury. The patient before you strides rapidly, his eye is fixed, his lips pale, he overturns everything, nothing but must yield to his violence. His attitude is haughty, threatening: his silence, like his vociferations, inspires terror. He deals out blows: he is seized. He resists: a struggle follows; he is shut up. Alone, he rends his clothes, breaks up his bed, seizes the fragments and beats the door. From a corner of his cell he defies all who dare to enter. At the end of some hours or days he is fatigued, and seeks repose.

In the midst of all these acts, the bodily movements are executed with remarkable harmony and suppleness. The muscular force is often increased to an extraordinary degree.

In mania, as in melancholy and ecstasy, the sleep is imperfect and irregular. Often the patient sleeps during the day, and sings and shouts at night. This condition reacts strongly upon the intellect, which it obscures. It is said that the patient understands his position, unless it be at the outset of his illness. He cannot believe in a disease of the mind.

The maniac is credulous, easily deceived: his judgment is enfeebled. Most frequently the aptitude for work is diminished, or absent. It only appears when the disease is on the decline.

The appetite increases; it is sometimes voracious: this is a pathognomic symptom of mania. The increase of the appetite is almost always the indication of a coming maniacal attack. Some, however, refuse obstinately to eat.

Some maniacs drink copiously. In the greater number the stools are regular; but diarrhœa and constipation are observed. The urine exhibits nothing remarkable in tranquil mania. During the maniacal attacks, in cases of great agitation, the urine has often an inflammatory aspect; there is a deposit; the colour is very deep, and resembles the urine proper to the crises of gout. In some maniacs the attacks are announced by an incontinence of urine; when the disease diminishes in intensity, this involuntary flow ceases. In chronic cases, and especially in maniacs advanced in years, this symptom is of most unfavourable augury, indicating the transition from mania to incurable dementia.

Examine the pulse in the greater number of these maniacs, and you will find it of remarkable celerity. Most frequently the cerebral excitation may be measured by the rapidity of the pulse. Occasionally it is slow, as in some cases of melancholy and ecstasy, but then it presents a particular rhythm; every pulsation, even when the cardiac contraction recals the physiological condition, presents a certain vivacity of a convulsive character. Seldom is there fulness or hardness in the pulse.

In recent cases, the carotid and temporal arteries beat with violence; the face of the patient is flushed.

In chronic cases the face and lips are mostly pale.

In recent cases in young and vigorous subjects the skin is moist, and even in the middle of the winter we are astonished to find it warm. Sometimes it is bathed in sweat, especially when the disease advances by fits.

There has been observed in insanity, and chiefly in mania, a particular odour from the skin, which has been compared to that of the urine of mice. M. Jacobi denies the existence of this specific odour; he attributes it to want of cleanliness. I can give you the most positive assurance that it is in many cases a reality.

Often the maniac grows thin; and frequently also his adipose tissue becomes loaded with fat, as soon as convalescence is observed.

In the generality of acute cases, the catamenial flow is suppressed; but it is sometimes regularly continued in chronic cases.

*Progress of the Disease.*

Mania may be continued, remittent, intermittent, periodical. It may recur at long intervals. It is acute or chronic. It is primitive when it arises apart from other phenomena. It is secondary when it succeeds to the functional phenomena.

Among the precursory symptoms may be observed instability of character, a disposition to embark in rash enterprises, to change one's condition, to overthrow to-day the project of yesterday.

The disease may commence by dreams. The patient thinks he sees torrents, precipices, blood, flames; he fancies he is pursued by robbers, by gendarmes.

Sometimes it is announced by pains in the temples, forehead, or occiput, which disappear in a few days.

Sometimes the symptoms begin in the chest, by a feeling of oppression in the region of the heart, by spasms, palpitations, trembling of the hands and arms, and lips.

Occasionally the disease seems to radiate from the abdomen; sometimes there are violent colic pains; the tongue is loaded with a yellowish coating; there is loss of appetite—or there may be vomiting, prostration; it might be supposed that the patient was on the eve of a serious malady.

Very often, and especially in periodic mania, the skin is the seat of an eruption, partly erysipelatous, partly roseolate.

The patient refers to his head an uneasiness which he cannot define. "It is odd," he says; he puts his hand on his head and cannot explain what he feels;

"I am driven in different directions;" "I hear bells;" "I hear voices." "Singular ideas come into my head." In a few hours his whole face is decomposed; he would not be recognised. At the end of some days he sleeps a little; he is better in the morning. He is still better in the evening; but soon a new attack breaks out. The disease grows distinct; the patient is irritated against those who surround him. A fresh calm takes place, another attack appears. Soon there are nothing but remissions, which vanish as the maniac breaks out into cries and vociferations.

In some cases the invasion takes place without prodromata; it is sudden and violent.

The patient thus advances by starts towards a gradually increasing perturbation.

Mania terminates in different manners in health: by a temporary suspension; by prolongation; by an indefinite chronic condition; by a multiplication of its phenomena. It may end in a transformation into melancholy; into ecstasy; into delirium; into dementia; or by a manifestation of other diseases, as diseases of the encephalon, of the chest, of the abdomen; of febrile diseases; and by death.

In melancholics we have found exaltation at the approach of convalescence; in mania we find the reverse.

Mania, like melancholy, recurs by periodical returns; this is true of more than one-third of the number of maniacs. I do not think there is any regularity in these manifestations. It is especially in cases connected with epilepsy that the greatest regularity is observed.

The greater number of maniacs recover; according to my estimate, seven-tenths are restored to health; but sometimes this is only temporary.

In almost all the maniacs whose illness is protracted, there is observed a kind of cachexy, an emaciation, a pallor of the face. It would seem as if the mass of the blood had diminished, and that its colour had changed.

When recovery does not take place, the mania remains chronic, or changes its character, becomes associated with dementia, or passes entirely into this form, which is pre-eminently marked by a great incoherence of ideas.

I do not remember ever to have seen pure apoplexy in the course of mania.

Local paralysis, regarded as an accidental symptom, has been but seldom observed. The association with general paralysis is frequent.

Sometimes in the course of hyperphreny there supervenes a general and sudden prostration.

Death appears suddenly and unforeseen; this is especially the case in acute mania. But in many cases death is the consequence of a gradual extinction of strength, brought on by a marasmus which I will call cerebral.

### Tenth Lecture.

*Of the alienations which may be comprised under the denomination of folly** (*folie*).—I have now to bring before you an order of phenomena of unusual appearance, which often present a special analogy with certain acts committed voluntarily with the intention of mischief.

These forms of disease have been but vaguely described. They have been referred to monomania, melancholy, and even to mania. I have already said

* However awkward the word may appear, we have thought it expedient to translate the word "*folie*" into "*folly*." The author attaches a peculiar pathological signification to the French term; and if that signification be, in like manner, extended for the occasion to the English one, our task of rendering the ideas of M. Guislain upon this subject will be facilitated. We must admit, however, that the compound "*monofolly*" has a strange sound. Coleridge apologised for the introduction of the word, psychology.

that it was my intention to give precision to the term "folly." I wish to constitute it into a distinct morbid genus.

It comprises various types: simple and special vesania; general and compound affections.

I shall call the first *monofollies;* the others *polyfollies.* However strange the association of this Greek root and French word may appear, I venture to propose it.

Several *monofollies* are extremely rare, so much so, that many very aged practitioners may have witnessed but few of these morbid forms.

It is not the exaltation of the intellectual phenomena that you will find at the root of the disease, as we have done in mania. We have to remark in folly, acts impressed with the character of oddity, eccentricity, sometimes of excessive cruelty—acts executed with deliberation in the absence of all motive or real passion. It is said, it is generally believed, that the insane who commit these acts proceed designedly and in consequence of an internal deliberation. Most frequently there is nothing of the kind. The idea remains healthy, and commonly has nothing to do with these vagaries. The disease represents a monomania of actions rather than a monomania of delirious conceptions. The patient is urged on, he knows not how or why. His will seems principally affected; not his will of passions, but his will of irreflective actions, his impulsive will.

Hence this kind of disease has been called *instinctive monomania*, the *madness of action, impulsive alienation, extraordinary impulse.* The absence of motive is not an exclusive fact in this alienation. But the intervention of a morbid thought, this complication of this form of insanity, is not constant; it does not constitute a fundamental element in this vesania. It is an active passion, in which the patient is driven irresistibly to execute deeds of a capricious will, and which do not bear the character of a true passion acting and reacting. Innumerable facts prove that the most singular and eccentric acts may be manifested without any perceptible disorder of the conception or of the imagination. Starting from this, Prichard has assigned to all the instinctive extraordinary impulses a place in his scheme of *moral insanity*.

The term *moral insanity* is not a happy one. It represents a mental disease, incomplete, in a rudimentary state, at least according to the opinion commonly enunciated; it often constitutes the initial form, the prodromic period of an alienation to become more complete hereafter. Folly, then, may constitute one of the forms of moral insanity; but this, as you have already seen, may also be either a melancholy or a mania; it is the absence of delirious ideas which gives to moral vesania its pathognomic colours.

Folly, therefore, is allied to the special impulses having a character of morbid irresistibility. Certain species and varieties of this morbid genus have been described under the denomination of destructive monomania, homicidal monomania, pyromania, &c.

Our predecessors recognised a variation of this phrenopathy, and they appreciated it better than the moderns: they described it under the name of morosity, from *morio*, buffoon, fool. They even created an *alienatio morio*, a *mania morio*, a folly in which grotesque actions predominate.

I therefore establish a distinction between mania and folly. In folly you will observe oddities in the actions, rarely a passionate exaltation. Most frequently the progress is slow and insidious. In mania, it is exaltation, animation, which characterise the disease. The maniac is loquacious, quarrelsome, and aggressive. In the fool (*fou*), the expression of the physiognomy is usually normal. His conversation is not remarkable for exuberance of words. You would call this man serious, quiet, taciturn. Patients affected with folly produce a totally different effect upon the crowd from that arising from the observation of maniacal acts, of an ecstatic, of a melancholic.

On a close consideration of folly, it seems to consist of reflexiform impulsions. It is not a convulsion, but in essence it resembles it. It is not a fitful muscular movement, but a vicious direction of volition.

On observing these singular patients, in discoursing with them, frequently nothing reveals a diseased mind; they are attentive, they conceive, calculate, measure probabilities and impossibilities; their memory is intact, they remember facts, persons, and dates.

In a crowd of situations which the moderns have designated as suicidal, homicidal, and other forms of monomania, the madman is no longer the representative of human force: he is under the dominion of his instincts.

There is a circumstance which deserves our attention, which is, that often the patient has the appearance of regarding the facts which concern him as if he were not the author of them; he does not trouble himself about them, or the consequences.

Why, you will ask me, insist upon these distinctions? It is because they possess a real utility in relation to prognosis. The characters of mania, more essentially primitive, more violent in their course, are also of more favourable augury. The characters of folly, on the other hand, less often initial, rather secondary, slower in their development, and more insidious in their progression, inspire me for the most part with exceeding mistrust, and are far from cheering in reference to the curability of the patients.

I have known patients who have said to me, "Something, I know not what, an electric force, perhaps, compels me to take up this book, or other object, and to throw it to the ground. I *must* lift up my arm. I *must* move that table, this chair. I undress myself without knowing why; I *must* act in opposition to my intentions." Others say, "There is in me some one who is not myself—who drives me and forces me to act."

I cannot venture to affirm that the unmarried are more subject to this affection than the married; but I have reason to believe that the predisposition is stronger in the former.

The symptoms proceed in the form of crisis, of fits, which are mostly manifested in an explosive manner. I am in the habit here of calling them rockets, from the sudden nature of their manifestation.

When these fits appear, they are usually accompanied by anxieties, vague terrors, hallucinations, agitation, and many acts which we also recognise in epileptics before the explosion of the convulsions.

At these moments, the patient kills his children, his father, his friends. He drinks boiling water. He throws himself from a height, or hangs himself. He takes a knife or razor and cuts his throat. He is rarely seen to destroy himself by the aid of fire-arms.

It is to one of these situations that some observers have given the name of *mania brevis*, and hence *mania instantanea*.

This impulsion has not always murder for its object; it occasionally breaks out in singularities, in childish oddities, in species of momentary distractions. These patients are for the most insensible to all stimulants. In the depth of winter, one of our patients perceives a finger numbed with cold. Having occasion to use a knife, he cut off this finger at a joint. He always said that he felt not the slightest pain during the operation.

A few days ago, I offered some snuff to a suicidal madman; he was in one of his lucid moments. "Is it not strange," he said, "that when I am well, a grain of snuff is enough to make me sneeze five or six times: now I take any quantity, and I cannot excite the sensibility of my nose; I do not sneeze at all?"

During the crisis the pulse is sometimes very slow, sometimes very quick. The skin is often bathed with sweat. But we cannot recognise in these symptoms the fits of an intermittent fever; it is rather neuralgic or convulsive fits that we should assume as points of comparison.

In this kind of phrenopathies, the *visceral functions* are scarcely influenced in a permanent manner, as is the case in melancholy, ecstasy, and mania.

Fantastic impulsions may also be developed in the course of almost all the phrenopathies. Thus the refusal to eat is present in melancholy, the whim of dressing up in odd costumes in mania, and automatic acts are seen in dementia.

Regarded as an elementary form, folly, therefore, is not a grief, an anger, nor a disturbance of the reason. In this vesania, the morbid impulsion seems to start from other centres than those in which the passions are developed, and where the ideas reside.

*Of the different forms under which folly may present itself; their associations with other phenomena. Special follies.*—We have here patients impelled by an irresistible desire to bite, or tear with their teeth, everything in their way. We shall name these the *biting fools.*

This condition seldom belongs to an isolated impulsion; it generally forms with other kinds of vesania a compound alienation. We have here several *rending* madmen. There is a propensity to tear or cut everything to pieces. This may be a real *rending monophreny,* when the desire of destruction presents a dominant character.

We might call *mutilators* those madmen who turn against themselves their irresistible want to mutilate living beings. It is only observed in exceptional cases.

*Self-murderers.*—In a nosographical point of view, I recognise:—

A. A pure suicide, a *suicidal monofolly,* consisting in a blind, irresistible impulsion.

B. A *suicidal monomania,*—that is, a mania with suicide, when the patient destroys himself in a fit of rage.

C. A *suicidal monomelancholy.*

D. A *delirium with suicide,* as we shall see further on.

Suicide, as I understand it, may constitute a radical symptom, an essential disease; or else it is only an epiphenomenon, appearing in the course of another vesania. This latter form is most frequently observed in melancholy. Melancholy, moreover, is at the bottom of almost every form of suicide. Suicide may be manifested in dementia. It may also present itself, without the slightest disorder of the ideas, in *suicide without delirium.*

Suicidal epidemics have been observed.

In some cases, the suicidal desire is constant. It may be remittent, intermittent, or periodical. It may be propagated by the influence of imitation. The species of moral contagion which distinguishes this affection has long been recognised. Esquirol and Falret first called attention to it.

*Clinical examination of a suicidal patient.*—How is the suicidal tendency observed in this patient? I will tell you. After some months of sadness the affection broke out suddenly: the patient was as if hunted; he is still driven by an internal force. He mostly speaks to you with perfect sense. He talks of his disease, and explains how he is carried away in spite of himself. You heard him say, "Whilst I am talking to you, I feel my head working." Soon, he will speak no more, he will look at you with a pre-occupied air, and will appear quite beside himself. The fits last some hours; he comes to himself, remains calm for some hours longer, for a whole day, until the morbid agitations return, and finish by becoming continuous.

Have you observed the singular look of this patient, and that deeply-grave and serious expression spread over his countenance, the colour of his skin, the tension and the pallor of his lips? And then his conversation. There is nothing more striking than the integrity of his reason. Often these patients themselves request that all the precautions their condition requires should be taken. In the midst of all these symptoms there is frequently an oppression of the chest, which deserves all the attention of the practitioner. It is accompanied at times by excessive paleness, lividity, and a pulse remarkable by its slowness and fulness, and in certain cases by its extreme frequency.

The other day I asked the young man whom you see there—he is convalescent from a suicidal folly—if he had the consciousness of the first attack of his disease. Yes, he answered, perfectly. It began by a stifling, a pain at the bottom of the chest; the suffering was great; it cut short my speech; but it did not last long: it came back, however, and at every return, it seemed to me as if I could not see; everything around me disappeared; I heard nothing. I thought something dreadful was to be done to me, and I ran straight to the river. I did not feel the water, and what passed there I know not. I must have been picked up, since I am still alive.

The organs of the chest, therefore, play an important part in suicide; the heart often seems to be in a quite peculiar condition. The alterations of the heart, as I shall prove, the white spots on its external surface, the morbid granulations of this surface, the adhesions between the two laminæ of the pericardium, have presented themselves to my observation. I have been driven sometimes to seek some abnormal condition of this organ in its irritability, in its nerves, in its structure.

You may read with profit what Fr. Nasse has said concerning the influence of the heart upon mental diseases.

This condition is sometimes connected with the critical age, the appearance or suppression of the catamenia, or of hemorrhoïds, with a gouty cachexy, with an abnormal constitution revealed by a dark brown complexion, dark rings round the eyes, the projection of the belly, sluggish bowels, dark-coloured urine, and general thinness.

Suicide is often associated symptomatically with all the kinds of destructive folly. But a profound moral grief is almost always detected.

In Belgium this vesania is very rare. In the united establishments of Ghent it is not observed five times out of 100 admissions, always excepting those who suffer themselves to die by refusing food.

In France, out of 34,000,000 of inhabitants, there were 30,000 suicides in eighteen years.

The *homicidal monomania* of Esquirol is, nine times out of ten, the effect of a motiveless impulsion, which drives the patient to commit murder.

Homicidal madmen believe that they must act so; they kill, they say, because they are driven to it.

In a diagnostic point of view, it is essential to mark the distinction that exists between the homicidal hyperphreny and the folly of the same name. In the first case, the patient reveals in his features, in his attitude, all the characters of an overflowing passion of rage; he howls, overturns, destroys; his eye is on fire. In homicidal folly, it is quite different. We behold a patient who is taciturn, anxious, pale, indifferent, acting without anger, without fury, but who evinces the marks of an irresistible impulsion.

Homicidal folly may be a simple vesania.

But can a man, without presenting any preludes of illness, suddenly be carried beside himself, and cut off heads, arms, burn, strangle, without offering any other symptoms than a morbid perversion of the impulsive will?

Reason refuses to believe in such a state, and yet eminent men, among others Esquirol, assure us that such cases are real, but that they are rare. For my part, I have not yet met with destructive monophrensy without accessory symptoms.

During the fits, homicidal folly becomes complicated with a peculiar alteration of the features, and an extreme acceleration or retardation of the pulse.

Most frequently this alienation is a compound state, and is associated with transitory delirious ideas. The patients believe themselves inspired, they hear voices, entertain fears, and nourish hatred against their best friends.

Homicidal folly is rarely a transitory phenomenon; it is generally permanent.

It is in the category of destructive madmen that we must class certain patients, to whom I shall give the name of *Necrophilists*.

Mental pathologists have adopted as a new form the case of the sergeant Bertrand, the spoiler of graves, of whom the journals have recently spoken. But the ancients, in speaking of lycanthropy, have cited examples with which this case may be connected.

There is a variety of destructive folly, the *incendiary monomania* of Esquirol, to which Marc has given the name of *pyromania*, and which we will call *pyro-folly*.

This kind of vesania is rare; but there are at this moment three patients here, transferred from the prisons, presenting this affection; they had been accused before the tribunals for arson, and sent here as insane.

This folly is very rarely seen as a partial alienation; it is generally associated with other pathological elements.

### Eleventh Lecture.

We cannot avoid recognising in the patients who have been the subject of our study, an abnormal, diseased condition of the power which commands the actions. It is chiefly the will that is affected.

In the patients whom we shall see in the sequel, this condition of the impulsive forces will be found under other forms, with other results, but always preserving the eccentric, irreflective, unreasoning character, which I have said to be the fundamental symptom of the pathogenic gamut of folly.

Some fools are obstinate to a degree which cannot be conceived by those who have not been in contact with them. They refuse to change their linen, to sleep in their beds, to wash,—indeed, they resist everything they are asked to do. This is the *folly of opposition*.

The dumb constitute a remarkable type of the insane. One of our female patients obstinately refused to speak for three years: through refusal to eat, a state of marasmus had slowly come on. The sister Sylvia, the matron who had charge of her, said to her: "You may keep silent, if it suits you; but if you persist in not speaking, you will not live long: call me when you feel the approach of death." Shortly after, in the middle of the night, she awoke the sister, who was sleeping in the same room, crying out: "Come, come; I am dying." In a few minutes, she expired.

Sometimes there is not an intellectual incapacity but a caprice, a morbid whim.

Phrenopathic dumbness presents different varieties. I often observe it succeeding a long period of incubation, characterised by grief. Sometimes it is met with as a transitory symptom in a group of other phenomena. I have recognised it as the type of a monomania which I will call *mutomonofolly*.

There are important distinctions to be drawn in regard to this vesania.

*a*. In incomplete ecstasy, there is an impossibility of speaking.

*b*. I have known men, who, after a typhoid fever, have been affected with an impossibility of speaking.

*c*. In melancholy, patients frequently will not answer when they are spoken to.

*d*. It is sometimes difficult to distinguish the phrenopathic dumbness from deaf-dumbness. But the state of the hearing assists the practitioner.

*A fasting madness.*—The refusal to eat is a symptom often met with in mental alienation. It is, so to speak, a variety of the preceding forms of opposition, and the refusal to speak. The refusal to eat is in every case a serious indication. It carries many patients to the grave by inducing a special affection, which, as I have shown, is a pulmonary affection connected with a general vitiation of the blood.

Once only have I found a state of complete *mono-sitophobia* apart from all combination with other symptoms of alienation. I am speaking of a young person, who, in consequence of a moral cause, a wound to her self-esteem, evinced a repugnance for every kind of food. This state degenerated into absolute refusal to eat, and finished by exhibiting itself under the form of a partial mental alienation. It is one of the most curious cases I have ever witnessed. For a long time the condition of this patient was looked upon as the result of an affection of the stomach, an anorexia. Her unconquerable obstinacy in refusing nourishment, her progressive emaciation, at length opened the eyes of her parents, and she was sent to me. The success of a moral treatment energetically pursued, followed by the recovery of the patient, testified to the justness of the diagnosis.

I shall have an opportunity of showing you the reasons which lead me to believe that in the refusal to eat, the eighth pair is morbidly affected.

This vesania is rarely a simple affection.

We have here some *daubing* madmen, who, if allowed, would do nothing but daub the walls with grotesque figures.

There are *hiding* monomaniacs, who conceal everything that comes in their way. There is also a mania for theft: this resembles criminal theft. *Clepto-folly* is usually observed as an element of association, or a transitory phenomenon in the alienations with exaltation of the passions. It also characterises the decadence or obliteration of the faculties of the intelligence; it is met with as an epiphenomenon in dementia, in idiocy, and epileptic convulsions. This morbid form is never observed in melancholy or in ecstasy.

Here is a madman who is incessantly digging the earth in the garden. I have entered his disease under the name of *talpafolly*. His proceeding resembles the action of the mole. It is not the first time that I have observed this phenomenon.

We have here fools addicted to *oratory, declamation, monologue*, and *dialogue*. Some affect to speak languages they do not understand. I have met patients who always repeated twice the same phrase.

There are *shrieking* and *howling* madmen. Others imitate the *song of birds*, the *mewing* of cats, or the *barking* of dogs.

These affections have often an intimate relation with hysteria. They even constitute diseases of transition, mixed conditions, phrenopathies on the one hand, a subconvulsive state on the other.

*Gesticulating madmen.*—Sometimes folly constitutes almost a variety of chorea. I have brought before you some patients who perform without ceasing the most singular movements of the mouth, tongue and face. This is *mimo-folly*. The patient to your right is a striking example: for four years that he has been here, he has never left off executing a fantastic contraction of the muscles of the left cheek.

Sometimes they preserve a crooked attitude. It is in vain that you offer them a chair, a stool, or a bench.

There is in the asylum a girl who for eight months has never opened her eyelids.

*Fantastic automatism* is often the prelude or the accompaniment of dementia.

When in the course of a mania or a folly, the intellectual functions undergo insensibly a subtraction of energy, when there is a progression towards dementia, there is often observed a quite peculiar excitation in the gait, in the gestures, in certain acts. These acts, these gestures appear under an automatic form. There is in reality an antagonism between what have been called for some time past the instinctive acts and the intellectual acts. In proportion as the latter decline, the former become exalted.

It is then especially that we remark the balancing of the body, the act of netting, a species of carphology; the fancy for stripping naked.

There are fools who present the phenomena of hysteria, of catalepsy, of epilepsy. Epilepsy, however, belongs more especially to mania than to folly.

---

### INSTITUTIONS FOR THE INSANE IN PRUSSIA, AUSTRIA, AND GERMANY.*

